# Genetics and epigenetics of autism spectrum disorder—current evidence in the field

**DOI:** 10.1007/s13353-018-00480-w

**Published:** 2019-01-10

**Authors:** Barbara Wiśniowiecka-Kowalnik, Beata Anna Nowakowska

**Affiliations:** Institute of Mother and Child, Warsaw, Poland

**Keywords:** Autism spectrum disorders, Copy number variants, Single nucleotide variants, Heritability, Synapse

## Abstract

Autism spectrum disorders (ASD) is a heterogenous group of neurodevelopmental disorders characterized by problems in social interaction and communication as well as the presence of repetitive and stereotyped behavior. It is estimated that the prevalence of ASD is 1–2% in the general population with the average male to female ratio 4–5:1. Although the causes of ASD remain largely unknown, the studies have shown that both genetic and environmental factors play an important role in the etiology of these disorders. Array comparative genomic hybridization and whole exome/genome sequencing studies identified common and rare copy number or single nucleotide variants in genes encoding proteins involved in brain development, which play an important role in neuron and synapse formation and function. The genetic etiology is recognized in ~ 25–35% of patients with ASD. In this article, we review the current state of knowledge about the genetic etiology of ASD and also propose a diagnostic algorithm for patients.

## Introduction

Autism spectrum disorders (ASD) is one of the most prevalent group of neurodevelopmental disorder that affects around 1–2% of the population with the average male to female ratio 4–5:1. ASD is characterized by social interactions and communication deficits, repetitive and stereotyped behavior (Lai et al. 2014; Baio et al. 2018). Furthermore, approximately 31% of patients with ASD also present intellectual disability (ID) (Baio et al. 2018), and 20–37% suffer from epilepsy (Canitano 2007; Yasuhara 2010). In addition, the epileptic EEG abnormalities can often be found in autistic children, even without the incidence of seizures (Yasuhara 2010). Moreover, children with ASD often present other psychiatric and medical conditions including anxiety disorders, depression, attention deficit hyperactivity disorder (ADHD), sleep disorders, and gastrointestinal problems (Valicenti-Mcdermott et al. 2006; Richdale an Schreck 2009; White et al. 2009).

Essential autism spectrum disorder is diagnosed in approximately 75% of the ASD patients. The prevalence of this type of ASD is in approximately 35% of the siblings and about 20% of cases have positive family history for ASD. While, syndromic ASD occurs in approximately 25% of patients. In these individuals, autistic features co-occur with dysmorphic features or congenital anomalies. Moreover, the sibling recurrence risk is lower (4%–6%) than in essential ASD, and family history is less frequent (9%) (Elsabbagh et al. 2012).

Although it was shown that ASD have a complex multifactorial etiology, twin studies proved a strong genetic contribution. The concordance rate of autistic disorders in monozygotic twins is 70–90% while in dizygotic twins is up to 30% (Rosenberg et al. 2009; Hallmayer et al. 2011; Ronald and Hoekstra 2014) and 3–19% in siblings in general (Ozonoff et al. 2011; Constantino et al. 2013). Furthermore, twofold greater concordance among full siblings than in half siblings provided the evidence that genetic factors play an important role in the development of ASD (Constantino et al. 2013). Nowadays, the genetic etiology is recognized in ~ 25–35% of patients with ASD.

Even though the background of ASD is only partially understood, the great number of symptoms observed in patients with autistic disorders suggests that ASD have multiple etiological factors, both genetic and environmental. Furthermore, gene-environment interaction can lead to epigenetic abnormalities and cause alterations in the brain anatomy and connectivity characteristic for ASD (Schaevitz and Berger-Sweeney, 2012).

## Chromosomal abnormalities

Currently, it is estimated that classical karyotyping techniques can reveal chromosomal aberrations in approximately 2–5% of ASD individuals (Devlin and Scherer 2012, Liu and Takumi 2014). Large unbalanced karyotypic abnormalities are found more often in ASD cases with associated dysmorphic features. Structural chromosomal alterations have been reported for every chromosome and include deletions, duplications, inversions, translocations as well as marker chromosomes. Most of structural aberrations are rare and their causal role in ASD is not clear, but few of them are recurrent (Castermans et al. 2004). The most frequent chromosomal abnormality detected in 1–3% children with ASD is maternally derived 15q11q13 duplication, with variable size (Hogart et al. 2010). Many genes in this chromosomal region have essential functions in the brain, such as *GABRA5* and *GABRB3* (GABA receptors), *UBE3A* and *HERC2* (components of the proteasome complex) and *SNRPN* (ribonucleoprotein peptide N) as well as *CYFIP1* (the FMRP interacting protein) (Menold et al. 2001; Nishimura et al. 2007; Bucan et al. 2009; Puffenberger et al. 2012). Other chromosomal abnormalities identified in ASD patients include aneuploidies: 21 (Down syndrome), X (Turner syndrome, Klinefelter syndrome, XXX syndrome), and Y (XYY syndrome) (Devlin and Scherer 2012).

## Copy number variations

Array comparative genomic hybridization (array CGH) allows to detect chromosomal microdeletions and microduplications that are too small to be identified by karyotyping. Research studies have shown that clinically relevant CNVs (copy number variants) invisible in karyotype analysis are detected in 7–14% of patients with idiopathic ASD (Rosenfeld et al. 2010; Roberts et al. 2014; Geschwind and State 2015). Rare de novo CNVs are identified more frequent in individuals with sporadic ASD then in autistic cases with affected sibling. Sebat et al. (2007) conducted one of the first studies which proved association of the de novo CNVs with ASD. They identified such CNVs in 10% of patients from simplex families and in 3% of individuals from multiplex families, and in only 1% controls (Sebat et al. 2007). This suggests that rare de novo CNVs may be significant risk factors for ASD particularly in patients with sporadic disorder. Similar results were obtained in further studies that identified de novo CNVs in 5.8–8.4% of sporadic ASD (Marshall et al. 2008; Levy et al. 2011; Sanders et al. 2011).

Copy number variants can be divided into recurrent and nonrecurrent CNVs which are correlated with the presence of specific elements of the genome structure that predispose to their occurrence. Recurrent CNVs shared a common size and breakpoints, and are caused by non-allelic homologous recombination (NAHR) between low-copy repeats (LCRs). LCRs are DNA blocks of 10 kb to several hundreds of kilobase in size and have 95–97% sequence identity. This high degree of sequence similarity predispose to NAHR and result deletions and/or duplications. Whereas nonrecurrent CNVs usually arise by non-homologous end joining (NHEJ), fork stalling and template switching (FoSTeS) and microhomology-mediated break-induced replication (MMBIR). NHEJ is the mechanism used to repair double-stranded DNA break (DSB) but leaves a so-called “molecular scar” (microhomology or insertion) in the novel junctions. FoSTeS i MMBIR are replication-based mechanisms (Gu et al. 2008; Hastings et al. 2009).

Most CNVs detected in ASD individuals are sporadic and nonrecurrent which prove genetic heterogeneity of these disorders (Shen et al. 2010). The most common recurrent ASD-associated CNVs are the approximately 600 kb microdeletions and microduplications at the 16p11.2 region that are identified in about 1% of ASD individuals (Weiss et al. 2008; Fernandez et al. 2010) (Table 1). Common phenotypic feature in patients with the 16p11.2 deletion is macrocephaly whereas patients with duplication have microcephaly. Another recurrent CNVs detected in ASD cases include 1q21.1, 15q13.3, 17p11.2, 22q11.2, 16p13.1 and microduplication of 7q11.23 (Moreno-De-Luca et al. 2013; Tropeano et al. 2013) (Table 1). Furthermore, microarray analysis revealed several nonrecurrent microdeletion including regions of 2p16.3, 7q22q31, 22q13.3, and Xp22 (Marshall et al. 2008; Prasad et al. 2012; Béna et al. 2013). Most of CNVs harbor numerous genes that mutually may contribute to the ASD phenotype (Doelken et al. 2013). The most frequently identified recurrent CNVs in patients with ASDs are listed in Table 1.

**Table 1:** Recurrent CNVs identified in patients with ASD

Locus	Clinical features associated with CNV
1q21.1 deletion syndrome	Mild to moderate ID, schizophrenia, mild dysmorphic facial features, congenital heart abnormality, microcephaly, cataracts
1q21.1 duplication syndrome	Mild to moderate ID, ADHD, mild dysmorphic features, macrocephaly, hypotonia
2q37 deletion syndrome	ID, dysmorphic facial features, brachydactyly
3q29 deletion syndrome	Mild to moderate ID, schizophrenia, mild dysmorphic facial features
7q11.23 duplication syndrome	ID, schizophrenia, abnormal brain MRI, variable dysmorphic features
15q11q13 duplication syndrome	Mild to severe ID, epilepsy, ataxia, behavioral problems, hypotonia
15q13.3 deletion syndrome	Mild to severe ID, epilepsy, learning difficulties, ADHD, variable dysmorphic features
16p11.2 deletion syndrome	Mild to severe ID, epilepsy, multiple congenital anomalies, variable dysmorphic features, macrocephaly, obesity
16p11.2 duplication syndrome	Mild to moderate ID, ADHD, microcephaly, dysmorphic features
16p12.1 deletion syndrome	Mild to moderate ID, ADHD, congenital heart defects, craniofacial dysmorphology
16p13.1 deletion	ID, schizophrenia, epilepsy, multiple congenital anomalies, dysmorphic features
17p11.2 deletion syndrome	ID, speech delay, hearing loss, sleep abnormalities, hypotonia
17p11.2 duplication syndrome	Mild to severe ID, congenital anomalies, dysmorphic features, hypotonia
17q12 deletion syndrome	Mild to moderate ID, schizophrenia, epilepsy, MODY, dysmorphic facial features
17q21.31 deletion syndrome	Mild to severe ID, epilepsy, structural brain abnormalities, musculoskeletal anomalies, dysmorphic features, hypotonia
17q21.31 duplication syndrome	Mild to moderate ID, microcephaly, hirsutism, facial dysmorphism
22q11.2 deletion syndrome	ID, schizophrenia, learning difficulties, multiple congenital anomalies, congenital heart defect, dysmorphic features
22q11.2 duplication syndrome	ID, schizophrenia, speech impairment, learning difficulties, heart defect, dysmorphic features, microcephaly

*ADHD* attention deficit hyperactivity disorder, ID - intellectual disability, *MODY* maturity onset diabetes of the young

## Incomplete penetrance and variable of expression

Some ASD-associated CNVs are inherited from an unaffected parent or are found in control populations which prove different penetrance of these CNVs. Moreover, the same CNVs are detected both in ASD cases and in patients with other neurocognitive disorders including mental retardation/DD, epilepsy, schizophrenia, bipolar disorder, and ADHD that suggest that the final phenotype depend on the occurrence of additional rare genetic (or non-genetic) factors (Girirajan and Eichler 2010).

There are some explanations for phenotypic variability in genomic disorders. First, size of the CNVs can be different, thus involve various genes and causes different phenotypes. Furthermore, it can be recessive mutation or functional polymorphism within the CNV region which influence on the phenotypic variability. A single nucleotide variant can be also in close proximity to the CNV thereby modifying the expression pattern of the genes in the CNV region (Girirajan and Eichler 2010). Another cause of incomplete penetrance of the CNVs is the “two-hit model.” It is estimated that 10% of the patients also carry another CNV or point mutation. The second variants in one individual could change the dosage for several genes and lead to difference in the severity and variability of clinical features. It is possible that one hit is sufficient to cause some clinical features but a second hit is responsible for more severe phenotype with intellectual disability (Girirajan et al. 2012).

## Monogenic syndromes associated with ASDs

Approximately 5–10% of ASD patients have co-occurring monogenic syndromes or disorders. The mutated genes are often regulators of the expression of a large group of other genes. The most common ASD-related syndrome is fragile X syndrome (FXS) diagnosed in about 1.5–3% of individuals with ASD. FXS is caused by mutations in the *FMR1* gene that regulates about 6000 mRNAs in the brain and plays an essential role in synaptic plasticity (Ascano et al. 2012) (Table 2). Another frequent ASD-related syndrome is tuberous sclerosis complex (TSC) which occurs in about 1% of patients diagnosed with ASD. Two causative genes, *TSC1* and *TSC2*, are inhibitors in the mammalian target of the rapamycin signaling pathway (mTOR) that is involved in the local translation in the central nervous system. Mutations in the *MECP2* gene are responsible for Rett syndrome that is found in an additional 1% of female ASD patients. The MeCP2 protein (methyl CpG-binding protein 2) is a transcription factor that regulates the expression of many genes in neurons (Liu and Takumi 2014). Moreover, mutations in the *PTEN* gene that indirectly repress the mTOR pathway are responsible for spectrum phenotypes including ASD with macrocephaly (Herman et al. 2007) (Table 2). Other examples of single-gene syndromes associated with ASD include neurofibromatosis type 1 (*NF1* gene), Duchenne muscular dystrophy (*DMD* gene), and Timothy syndrome (*CACNA1C* gene). ASD can also occur in some metabolic diseases such as phenylketonuria (*PAH* gene) and Smith-Lemli-Opitz syndrome (*DHCR7* gene) (Caglayan 2010). Several studies have proposed that another group of ASD related to monogenic disorders is caused by mutations in the mitochondrial DNA (mtDNA) and impairment of mitochondrial energy metabolism (Wang et al. 2016; Valiente-Pallejà et al. 2018).

## Genome-wide association studies

Linkage studies examine the co-segregation of chromosomal regions with the phenotype among multiply-affected pedigrees. Heritable variants investigation within ASD families is supported by the high sibling recurrence (Sandin et al. 2014). Nevertheless, linkage study has small potency for disorders with complex etiology and inherited variants of uncertain significance. Previous linkage study identified linkage signals in almost all chromosomes but the most replicated chromosomal region was 7q35 (IMGSAC 2001).

The genomes of people differ from each other in genetic variants called single nucleotide polymorphisms (SNPs). Despite that most of these variants are common and occur at least in 1% of the population, some of these SNPs may increase the risk of developing complex, polygenic diseases (Voineagu 2012). It is thought that single common variants contribute to genetic, heterogenous disorders by epistatic interaction but the studies’ results indicate that SNP have small effect size on ASD. Several large-scale genome-wide association studies (GWAS) found many significant markers associated with ASD but predominantly specific to a single study (Ronald et al. 2010, An and Claudianos 2016). Large phenotypic variety in ASD causes that many GWAS tried to find association between genetic variants and subphenotypes of ASD (Hu et al. 2011; An and Claudianos 2016). Hu et al. (2011) divided all ASD individuals into four subphenotypic groups and identified 18 significant SNPs. Whereas, when they combined all patients into one cohort and analyzed together, there was no association found between SNP and ASD. This study confirmed that it is important to analyze clinical homogeneous groups of ASD individuals in GWAS (Hu et al. 2011). In addition, the more complex the disease is, the greater the probability that many genes are associated and many different polymorphisms affect its heterogeneity; therefore, the greater cohort size must be tested. To date, cohort sizes tested in ASD studies were around 2000 families while in other common disorders such as epilepsy or schizophrenia, studies were performed on much greater numbers of cases (more than 26,000 and 30,000, respectively) (Wang et al. 2009; Weiss et al. 2009; Anney et al. 2010; Anney et al. 2012; International League Against Epilepsy Consortium on Complex Epilepsies 2014; Schizophrenia Working Group of the Psychiatric Genomics Consortium 2014). Considering the large phenotypic variety of autistic disorders, the sample sizes are too small to completely understand the mechanism of ASD.

## Genes associated with ASD

Mutations and CNVs implicate genes encode the proteins that play an important role in the chromatin remodeling (*CHD8*, *BAF155*), as well as synaptic cell adhesion molecules (Neurexin and Neuroligin families, *CNTN4*), neurotransmitters, scaffolding protein in synapse (*SHANK2* and *SHANK3*), and ion channel proteins (*CACNA1A*, *CACNA1H*, *SCN1A*, *SCN2A*). The proteins are also involved in the signaling pathways and neuronal networks linked to synaptic gene transcription and translation pathway (*FMR1*, *TSC1*, *TSC2*, *PTEN*, *NF1*, *CYF1P1*), ubiquitination pathway (*UBE3A*, *PARK2*, *TRIM33*), protein synthesis and degradation, and participate in the development, formation, and function of synapses and neurons (De Rubeis et al. 2014; Iossifov et al. 2014; Pinto et al. 2014; Cotney et al. 2015; Hormozdiari et al. 2015) (Table 2). Moreover, results of the studies indicate that an imbalance in excitation and inhibition synaptic inputs could explain deficits in social and cognitive functions present in ASD patients. Chromatin remodeling regulates gene expression and may influence the formation and differentiation of neurons (Ronan et al. 2013). Cotney et al. in the in vivo study showed that the *CHD8* gene is strongly associated with ASD (2015). This gene encodes the chromodomain helicase DNA-binding protein that regulates the expression of many other ASD risk genes in the human midfetal cortex (Cotney et al. 2015). Moreover, neuronal activity affects the post-translational modification of synaptic molecules and gene transcription that regulate synapse formation, maturation, and function. Mutations in genes involved in the activity-dependent pathways result in dysregulation of this network and cause of ASD (Ebert and Greenberg 2013). Voineagu et al. showed significantly lower expression of genes that play an important role in synapse formation and function in the autistic brain compared to the normal brain. The authors believe that one of the molecular mechanisms underlying ASD is the transcriptional and splicing dysregulation (Voineagu et al. 2011). Moreover, alteration of the proteins translation in the brain may lead to synaptic dysfunction and development of ASD (Gkogkas et al. 2013; Santini et al. 2013).

The next-generation sequencing technology allows precise analysis of the patient’s whole exome or genome and detects single nucleotide changes within one experiment. Thus far, genetic studies identified more than 1000 genes that contribute to ASD risk. De novo exonic mutations in the genes expressed in the brain have been identified in approximately 5–14% individuals with idiopathic ASD (Sanders et al. 2012; Iossifov et al. 2012; Lim et al. 2013).

The first whole exome sequencing study on ASD patients was reported in 2011 by O’Roak et al. The authors identified 11 de novo missense mutations in 20 trios, with idiopathic ASD. Four of them, in *FOXP1*, *GRIN2B*, *SCN1A*, and *LAMC3* genes, were classified as potentially pathogenic and probably were associated with ASD, ID, and epilepsy. The *FOXP1* gene encodes a transcription factor necessary for proper brain development. The *GRIN2B* gene encodes the glutamate receptor which binds the glutamic acid, the main neurotransmitter of the central nervous system. The *SCN1A* gene encodes a subunit of sodium channel, previously associated with epilepsy and indicated as the candidate gene for ASDs while the *LAMC3* gene is expressed in the cortex and limbic system (O’Roak et al. 2011).

Iossifov et al. (2012) sequenced exomes of the 343 simplex ASD families and found that gene-disrupting mutations appear twice as frequent in cases with ASD as in the cohort of unaffected siblings. Moreover, they claimed that many of autism susceptibility genes are linked with the fragile X protein. This supported association between ASD and the synaptic plasticity (Iossifov et al. 2012).

Further studies confirmed the contribution of de novo non-synonymous mutations in the higher risk of ASD. Sanders et al. (2012) identified such mutations in the genes expressed in the brain in 14% of patients. The results of their study as well as the studies of Neale et al. (2012) and Bernier et al. (2014) showed the association between *SCN2A*, *KATNAL2*, and *CHD8* genes and autism spectrum disorders. The *KATNAL2* gene plays an important role in the development of the nervous system (Neale et al. 2012; Sanders et al. 2012; Bernier et al. 2014). Moreover, the number of de novo mutations was positively correlated with the paternal age (Neale et al. 2012; O’Roak et al. 2012; Sanders et al. 2012).

In the next ASD study, Bi et al. (2012) identified mutations in seven genes, *ABCA1*, *ANK3*, *CLCN6*, *HTR3A*, *RIPK2*, *SLIT3*, and *UNC13B*, which play an important role in the neurodevelopment and synaptic function. Furthermore, they sequenced an additional 47 patients with ASD and found missense mutations in the *ANK3* gene in four unrelated cases. Previously, mutations in this gene were identified in individuals with schizophrenia or bipolar disorder, but the authors postulated that they also increase the risk for ASD (Bi et al. 2012).

Mutability depends on sequence and chromatin characteristics and also the regions flanked by segmental duplications may lead to a rise in recurrent mutations mediated by non-allelic homologous recombination (Michaelson et al. 2012). Michaelson et al. (2012) suggested that mutability is not the same throughout the whole genome and high mutability is characteristic for ASD genes.

Moreover, Turner et al. (2016) suggested that the small, often multiple CNVs and mutations disrupting putative regulatory elements may be one of the risk factors of simplex autism. They performed whole genome sequencing in 53 simplex families and identified more de novo and disruptive mutations and CNVs in the putative regulatory regions of genes that have been previously associated with ASD in probands compared to controls (Turner et al. 2016).

The most commonly identified mutations in patients with ASDs are listed in Table 2.

**Table 2:** Known genes associated with ASD

Gene	Clinical features associated with mutation
*CACNA1C*	Timothy syndrome features
*CHD8*	Macrocephaly
*CNTNAP2*	Mild to moderate ID, epilepsy, speech abnormalities, cortical dysplasia
*DYRK1A*	ID, microcephaly
*FMR1*	ID, Fragile X syndrome
*FOXP1*	ID, language impairment
*FOXP2*	Developmental verbal dyspraxia
*GRIN2B*	ID, epileptic encephalopathy
*MECP2*	ID, Rett syndrome
*NLGN4*	ID
*NRXN1*	ID, schizophrenia, mild facial dysmorphism
*PTCHD1*	ID
*PTEN*	Mild to moderate ID, macrocephaly, Cowden syndrome
*RELN*	Epilepsy
*SCN2A*	Epilepsy
*SHANK2*	Mild to moderate ID
*SHANK3*	Moderate to severe ID, severely impaired speech, schizophrenia, mild dysmorphic features
*SYNGAP1*	ID, epilepsy

*ID* intellectual disability

## Epigenetics

Epigenetic mechanisms modulate the chromatin conformation and regulate the expression of many genes without alterations in the DNA sequence (Schiele and Domschke 2017). Several studies proved the role for epigenetic dysregulation in the etiology of ASD. Mutations in genes encoding proteins implicated in epigenetic mechanism were reported in patients with ASD or ID. Duffney et al. (2018) reported a de novo mutation in the *HIST1H1E* gene encoding H1 histone linker protein in a 10-year-old boy with autism and intellectual disability. The H1 linker protein contributes to the organization of higher-order chromatin structures and regulation of gene transcription. The reported mutation cause lower protein expression and lead to develop ASD, ID, and behavioral problems. Subsequently, the authors reviewed databases of genes associated with ASD, and stated that 42 out of 215 genes causing ASD are directly involved in epigenetic modification of gene expression. Those genes encoded proteins that modify DNA or histones and regulate chromatin remodeling or the nucleosome assembly (Duffney et al. 2018).

One of the example of epigenetic regulation is DNA methylation. Numerous genome-scale studies revealed multiple alterations in DNA methylation in the brains of ASD individuals (Ladd-Acosta et al. 2014; Ellis et al. 2017). The largest study of meta-analysis of the peripheral blood samples in almost 800 autistic patients showed that 55 of analyzed CpG sites were associated with ASD (Andrews et al. 2018). Moreover, Berko et al. (2014) studied the ectodermal cell type of 47 ASD patients born to mothers at least 35 years old and defined various DNA methylation patterns compared to control individuals. This difference in DNA methylation was detected for the genes expressed in the brain and encoding protein that interacts with those involved with the development of ASD. The authors stated that epigenetic changes may be due to aging of the gametes or may occur in early embryonic life (Berko et al. 2014).

Furthermore, Atladóttir et al. (2012) suggested that epigenetic mechanisms can activate immune responses during pregnancy and also increase susceptibility to ASD. The results of this large population study implied that maternal influenza infection was related with twofold increased prevalence of having a child with ASD, prolonged period of fever during pregnancy was associated with a threefold increased prevalence of autism in children, and use of various antibiotics are risk factors for ASD (Atladóttir et al. 2012). Moreover the results of the study with a mouse model of maternal allergic asthma (MAA) suggested that alterations in maternal immune responses during pregnancy increase the risk of ASD in a child. MAA in mice caused changes in social and repetitive behaviors in the offspring. The researchers found differentially methylated regions in fetal microglia enriched for genes involved in immune signaling pathways and synaptic function as a result of MAA (Vogel Ciernia et al. 2017). These studies suggest that maternal immune responses can cause epigenetic changes and contribute to ASD development.

Recently, several studies explored the role of miRNA (microRNAs) in the etiology of ASD and demonstrated the alteration of the miRNA’s expression in ASD patients. MicroRNA is a small, 18–22 nucleotides in length, noncoding RNA molecule. Formation of these molecules occurs in several steps. First of them is a transcription that leads to formation of a precursor form called pri-miRNA (primary miRNA). Then, the first transcript is processed that leads to the generation of ~ 70 nucleotides long pre-miRNA. Both steps take place in the cell nucleus. The pre-mi RNA is transported to the cytoplasm where a number of processes lead to formation of a mature, functional, single-stranded miRNA molecule. These molecules control the expression of many genes through regulation of different mRNA activities resulting in inhibition of protein synthesis or mRNA degradation (Fregeac et al. 2016). The effects of miRNA deficiency during mammalian development were shown in studies of mice. The lack of Dicer ribonuclease, involved in pre-miRNA maturation, causes decrease of multipotent stem cells and death early in embryonic development (Bernstein et al. 2003).

About half of all known human miRNAs are expressed in the brain. It is believed that about 50% of human genes are regulated by these molecules and miRNAs control properly every functional pathways involving in the cell differentiation, proliferation, development, and apoptosis (Bernstein et al. 2003). Each miRNA can potentially regulate expression of numerous genes and each gene can be regulated by different miRNAs. There are different miRNA expression profiles in particular cells that enable precise modification of expression level of individual genes to the current needs of a certain cell. The studies of the animal models showed that deregulation of miRNA synthesis leads to the neurodevelopmental disorders (Bernstein et al. 2003; Hébert and De Strooper 2007; Davis et al. 2008; Kawase-Koga et al. 2009).

Recently, Kichukova et al. (2017) tested the expression profile of 42 selected miRNAs in serum of 30 patients with ASD. The results showed that relative expression levels of three miRNAs were evidently higher and the other five miRNAs were lower in ASD individuals compared to the control group. Next, analysis of genes that are regulated by these miRNAs showed that some of these genes are important for the CNS development. Therefore, the authors postulated that some of these miRNAs may play a role as biomarkers for ASD. The expression level of one miRNA was the same as in other post-mortem studies on brain tissue. The authors have also drawn a conclusion that miRNA expression dysregulation in the serum reflects abnormalities in tissues, and these miRNAs can be treated as biomarkers. However, further studies are needed to confirm if these eight miRNAs can serve as biomarkers for ASD (Kichukova et al. 2017).

## Genetic models of ASD

There are a few hypotheses which may explain the ASD etiopathogenesis. Based on the results of the GWAS, aCGH, and sequencing studies, we can conclude that there are common variants (CV) that increase the risk for disease development, but they are not sufficient for the disorder, and rare variants (RV) with moderate or large effect size (either CNVs or rare SNVs), both de novo and parentally inherited. The first proposed model is the rare variant–common disease (RVCD) hypothesis which suggests that one rare genetic variant with a significant risk causes ASD. This model is supported by the presence of de novo mutations in ASD patients that are not found in the control group, similarly in syndromic forms of ASDs where disruption of a single gene causes the disorder (Hatton et al. 2006; Khwaja and Sahin 2011). However, most of these syndromes demonstrate incomplete penetrance and variable expressivity for ASD. For example, only 30% of patients with the FraX syndrome have coexisting ASD, 10% do not show any autistic phenotype features, and some have only some ASD features (Iossifov et al. 2012; Harris et al. 2008). This variable expressivity and incomplete penetrance suggest that there is an additional factor (genetic, epigenetic, or environmental) that modulates the presence of ASD in individuals with the rare variants (Geschwind 2008). Moreover, rare variants are found in ASD individuals, unaffected relatives, and controls, for example maternally inherited 15q11q13 duplication, deletion of 22q13 and 2q37, CNV 17p11, and Xp22 (Vorstman et al. 2006). This makes it difficult to estimate which variants are pathogenic, which contribute to ASD, and which are unrelated to the clinical features.

An alternative hypothesis of ASD risk is the polygenic model in which combinations of various genetic variants lead to ASD. The first polygenic model the common variant–common disease (CVCD) proposes that many common genetic variants that occur in > 1% in the population collectively contribute to ASD. The large-scale GWAS showed that most of SNPs have a small effect. This implies that the importance of CVs is limited and many common variants are needed for ASD or another factor interacts with CVs and contributes to the disorder. The evidence supporting this model is that some relatives of ASD patients have autistic features, which suggests that only part of the CVs is sufficient for the occurrence of the endophenotype (Abrahams and Geschwind 2008). Whereas, the evidence against this model is the fact that in the GWAS studies only a small number of variants were found in more than one group of ASD individuals.

The second and third polygenic models assume that ASD is a result of rare and common variants combination. In the second polygenic model, ASD is a result of occurrence of a single RV among CVs. According to the third model, ASDs are caused by a combination of rare and common variants. These models are supported by existing rare variants inherited from healthy parents (e.g., 16p11.2) and some de novo CNVs are found both in patients and healthy people (Kumar et al. 2008; Bucan et al. 2009; Ben-David and Shifman 2012). Moreover, both rare and common variants have been identified in genes involved in neuron and synapse formation and development. Furthermore, some autistic features are present in relatives with identified genetic variants. These facts suggest that another factor contribute to ASD is required or some of these variants do not involve in autistic disorders.

In a fourth polygenic model, one RV predisposes to the disorder, while second RV causes the disorder or a greater pathology, compared to patients with only one RV. Therefore, this model is called “two hits model” (Berger et al. 2011). The results of many studies showed many examples of this thesis. Gau et al. (2012) found two aberrations (4q12q13 duplication, 4.5 Mb and 5q32 deletion, 1.8 Mb), one inherited from the mother and second from the father, in a boy with autism. None of these aberrations were in the database of genomic variants. The authors postulated that the presence of single CNVs is probably not enough to cause the disorder. The occurrence of two RVs in a patient with ASD suggests that the interactions between genes mapping within these aberrations may lead to the disorder. The support of this model is also the occurrence of rare variants inherited from parents and RV de novo found in healthy controls (Gau et al. 2012). The greatest support is for the polygenic model in which a combination of rare and common variants causes ASDs.

## Genetic diagnostic strategy

ASD is the heterogenous disorder and the genetic etiology is recognized in only about 25–30% of patients. This ratio is higher in ASD individuals with dysmorphic features, congenital malformation, seizures, or micro/macrocephaly.

ASD can be associated with some monogenic syndromes or metabolic disorders. Thus, the patient should have a medical evaluation for the presence of the clinical features characteristic for these diseases and molecular testing for mutations in the genes associated with these disorders should be performed. Among single-gene disorders for which ASD can be part of the phenotype spectrum, due to the incidence among ASD cases, mutations in three genes should be excluded. A screening for fragile X syndrome is recommended for all male patients even if they do not present any characteristic clinical features for FXS. Female testing for mutations in *FMR1* should be considered in case of X-linked pattern of inheritance of neurodevelopmental disorders, clinical features of fragile X syndrome, or premature ovarian failure in close relatives (Schaefer et al. 2013; Griesi-Oliveira and Sertié 2017). Moreover, all females with ASD and ID should be screened for mutations in *MECP2* which are associated with Rett syndrome (Schaefer et al. 2013; Griesi-Oliveira and Sertié 2017). Lastly, it is recommended to all individuals with ASD and macrocephaly to search for a mutation in the *PTEN* gene, which is responsible for harmatoma tumor syndromes that lead to macrocephaly and increased risk for tumorigenesis (Schaefer et al. 2013; Griesi-Oliveira and Sertié 2017). Next, due to the heterogeneity of CNVs, mostly submicroscopic in size, aCGH is recommended as the method of choice in cytogenetic diagnostics (Miller et al. 2010; Battaglia et al. 2013; Schaefer et al. 2013). In case of detection of copy number variant which is related to the phenotype of the patient, genetic counseling of the family is necessary. If the detected aberration has not previously been associated with autism spectrum disorders, the information about the function and expression of the genes that are located within the region, the size of the aberration, and its type (deletion/duplication) should be considered during estimating its clinical significance. In all cases, the origin of aberration should be determined in order to estimate the genetic risk in the family. Karyotyping is recommended only when there is a suspicion of aneuploidy.

In every case where aCGH analysis was normal, whole exome or genome sequencing could be considered, especially in patients with associated ID (Bourgeron 2015). NGS technologies are not a first-tier diagnostic test yet, because of the difficulty of the result interpretation. However, this may change due to an increasing number of NGS studies conducted on large cohorts of the autistic patients. The sequencing data from these studies probably will help in the interpretation of NGS results.

In each case, genetic counseling is necessary.

## Conclusions

Although chromosomal microarrays and sequencing technologies have greatly improved our understanding of the genetic etiology of ASD, there is still much more to discover. Despite of the identified hundreds of loci involved in ASD, the genetic variants classified as etiological factors are identified only in about 25–35% of the cases (Schaefer et al. 2013; Bourgeron 2015). Moreover, many genetic variants responsible for ASD are associated with other neurodevelopmental disorders or incomplete penetrance that makes it difficult to assess the genetic risk. Further studies are needed for better understanding of ASD etiology and also the different phenotypes among affected patients. It is expected that increasing amount of knowledge about etiopathogenesis of ASD and lower prices of technologies such as NGS will help to develop more accurate diagnostics and early detection of ASD. Possibly, it will allow to implement medical treatment based on genetic findings which now is not available for the majority of patients with ASD (Levy et al. 2009). Future work should focus on search for new strategies of treatment for autistic disorders based on the study of animal models with the same abnormalities as occur in ASD patients.
